# MicroRNA profile of extracellular vesicles released by Müller glial cells

**DOI:** 10.3389/fncel.2023.1325114

**Published:** 2024-01-18

**Authors:** William D. B. Lamb, Karen Eastlake, Joshua Luis, Najam A. Sharif, Peng T. Khaw, G. Astrid Limb

**Affiliations:** ^1^National Institute for Health Research (NIHR) Biomedical Research Centre at Moorfields Eye Hospital NHS Foundation Trust and UCL Institute of Ophthalmology, London, United Kingdom; ^2^Department of Global Alliances and Collaboration, Global Ophthalmology Research and Development, Santen Inc., Emeryville, CA, United States

**Keywords:** retina, Müller glia, extracellular vesicles, microRNA, neuroprotection

## Abstract

**Introduction:**

As with any other radial glia in the central nervous system, Müller glia derive from the same neuroepithelial precursors, perform similar functions, and exhibit neurogenic properties as radial glia in the brain. Müller glial cells retain progenitor-like characteristics in the adult human eye and can partially restore visual function upon intravitreal transplantation into animal models of glaucoma. Recently, it has been demonstrated that intracellular communication is possible via the secretion of nano-sized membrane-bound extracellular vesicles (EV), which contain bioactive molecules like microRNA (miRNA) and proteins that induce phenotypic changes when internalised by recipient cells.

**Methods:**

We conducted high-throughput sequencing to profile the microRNA signature of EV populations secreted by Müller glia in culture and used bioinformatics tools to evaluate their potential role in the neuroprotective signalling attributed to these cells.

**Results:**

Sequencing of miRNA within Müller EV suggested enrichment with species associated with stem cells such as miR-21 and miR-16, as well as with miRNA previously found to play a role in diverse Müller cell functions in the retina: miR-9, miR-125b, and the let-7 family. A total of 51 miRNAs were found to be differentially enriched in EV compared to the whole cells from which EV originated. Bioinformatics analyses also indicated that preferential enrichment of species was demonstrated to regulate genes involved in cell proliferation and survival, including PTEN, the master inhibitor of the PI3K/AKT pathway.

**Discussion:**

The results suggest that the release by Müller cells of miRNA-enriched EV abundant in species that regulate anti-apoptotic signalling networks is likely to represent a significant proportion of the neuroprotective effect observed after the transplantation of these cells into animal models of retinal ganglion cell (RGC) depletion. Future studies will seek to evaluate the modulation of putative genes as well as the activation of these pathways in in vitro and in vivo models following the internalisation of Müller-EV by target retinal neurons.

## Background

It has long been recognised that Müller glia, the radial glia of the retina, provide structural and key metabolic support to all retinal neurons (Reichenbach and Bringmann, 2020). As with any other radial glia in the central nervous system, they derive from the same precursors (Pacary et al., 2012), perform similar functions, and exhibit neurogenic properties as radial glia in the brain (Mori et al., 2005; Than-Trong and Bally-Cuif, 2015). In the zebrafish, Muller glial have been shown to regenerate the retina throughout life (Lenkowski and Raymond, 2014), and in the adult human retina, Müller glia with markers of regenerative cells with no evidence of regenerative functions have been identified (Eastlake et al., 2021).

Mammalian retinal neurons are incapable of spontaneous regeneration after injury, a characteristic that underpins retinal neurodegenerative conditions such as glaucoma, which is characterised by progressive death of retinal ganglion cells (RGCs), optic nerve damage, and subsequent loss of vision (Quigley, 1999; Tham et al., 2014). Current treatment strategies for glaucoma are based on controlling disease progression but are unable to repair existing neural damage and often fail to halt further neural loss (Sharif, 2023). Recent research in the stem cell field and the identification of Müller glia neuroprotective ability in the human retina have provided hope for the use of these cells in new therapies to maintain the viability and function of remaining neurons and potentially repair axonal damage (Singhal et al., 2012).

Previous studies have shown that Müller glia isolated from both the adult human retina and retinal organoids derived from pluripotent stem cells significantly improve visual function when transplanted into the vitreous of animal models of RGC depletion (Singhal et al., 2012; Becker et al., 2016; Eastlake et al., 2019). In these studies, improvement of visual function occurs despite the lack of integration of the transplanted cells, for which this effect has been attributed to the local release of neuroprotective factors by grafted Müller glia. On this basis, grafted cells could be considered “factories” for the long-term production and release of pro-survival molecules capable of protecting retinal neurons and preserving their function.

Secreted extracellular vesicles (EVs) comprising cytosol enclosed within a lipid membrane have been identified as a significant mode of intercellular communication. It has been proposed that EV-mediated transfer of molecules represents a hitherto under-appreciated mode of glial cell function within the nervous system (Budnik et al., 2016), and that EVs are a key component of the mechanism of action attributed to various stem cell-based therapies (Mendt et al., 2019). In particular, EVs have an innate ability to transfer genetic information and thus modulate gene transcription in target cells (Valadi et al., 2007; El-Andaloussi et al., 2012). Since the content of EV appears to be specific to the cells from which they derive, it has been suggested that these may represent a simple and sustainable source of neuroprotective molecules for application in retinal neurodegenerative conditions. Unlike whole cell transplants, soluble factors, or oligo-based therapies, EVs are non-replicative, highly stable, and easy to source (Kou et al., 2022).

Current lines of research focus on the ability of EV to transfer small non-coding RNA species, predominantly micro-RNA (miRNA), which are able to suppress the translation of specific mRNAs via the RNA-induced silencing complex (RISC) (Valadi et al., 2007). In the context of retinal neurodegeneration, previous studies suggest that the delivery of specific miRNAs via Schwann cell-derived exosomes into cultured neurons can promote neuritogenesis, and miRNA transfer has been suggested to be responsible for the neuroprotective efficacy of mesenchymal stem cell (MSC) exosomes in a rodent model of RGC depletion (Mead and Tomarev, 2017; Ching et al., 2018).

Given that Müller glia confer neuroprotection when transplanted into animal models of retinal degeneration (Becker et al., 2016; Eastlake et al., 2019), it is possible to suggest that miRNAs and proteins delivered via EV may be responsible for some or all of these effects. In the present study, we investigated the miRNA signature of Müller glia-derived EV by high-throughput sequencing and examined potential mRNA targets and putative pathways of activation for transcripts of interest.

## Methods

### Culture of the Müller cell MIO-M1

The Müller cell line (MIO-M1) established in our laboratory and derived from normal retinae (Limb et al., 2002) was used in the study. Each cell preparation was grown to a confluent monolayer on plastic flasks in DMEM containing 10% FCS. MIO-M1 cells used for EV isolation were between passages 20 and 30.

### Isolation of extracellular vesicles from Muller glial cell cultures

Extracellular vesicles were purified from Muller glial cell culture supernatants using ultracentrifugation in a protocol modified from that previously described by Gupta et al. (2018). Briefly, Müller cell cultures were grown to 80% confluence (to approximately 1.2×10^7^ cells) under standard conditions in 175 cm^2^ culture flasks and then washed three times in particle-depleted PBS. Cultures were then incubated for 48 h in 30 mL of vesicle-depleted media, which was collected, and centrifuged at 300 × *g* for 10 min. The supernatant was recovered, and the pellet was discarded. Centrifugation was repeated at 2,000 × *g* for 15 min and 10,000 × *g* for 30 min. The supernatant was then passed through a 0.22 μm filter (Corning^®^, UK) and then transferred to an ultracentrifugation tube, where it was layered on top of a 5 mL cushion of 30% sucrose solution (Sigma-Aldrich, MA, United States). The solution was then centrifuged at 100,000 × *g* for 120 min at 4°C, the supernatant was discarded, and the sucrose layer containing the vesicles was collected. Vesicles were washed in 10 mL of 1X PBS, passed through a 0.22 μm filter for a second time, and finally pelleted by centrifugation at 100, 000 × *g* for 120 min at 4°C. Depending on the downstream analysis used, the pellet was either re-suspended in 500 μL of sterile, particle-depleted PBS or processed for protein or RNA isolation as described below.

### Nanoparticle tracking analysis

The concentration and size profile of EV preparations were quantified by nanoparticle tracking analysis (NTA). Measurements were made using a NanoSight LM10 instrument equipped with a 405 nm LM12 module and EM-CCD camera (NanoSight, Malvern Panalytical Ltd., Malvern, UK). For each preparation, samples were diluted in particle-depleted PBS to obtain an average of 30–60 particles per frame, and a total of five videos of 30 s were taken for processing. For analysis, specific settings used were camera level 12, detection threshold 3, and blur size “Auto.”

### Transmission electron microscopy

Droplets (50 μL) of EV suspensions were adsorbed onto formvar/carbon-coated grids (Agar Scientific Ltd., Essex, England) for 20 min, before 3 × 30 s washes in droplets of particle-depleted DPBS. EVs were then fixed in a 1% glutaraldehyde (v/v) solution (Merck, Darmstadt, Germany) for 10 min at room temperature before a second round of washing. Negative staining was performed by a 15 min incubation in a 2% uranyl acetate solution (w/v) (Agar Scientific Ltd., Essex, England), after which excess stain was blotted and grids air-dried. Imaging was conducted on a JEOL-101100 kV transmission electron microscope (JEOLUSA, MA, USA) with image capture by a Gatan Orius digital camera. Micrographs were exported and processed using ImageJ software.

### Western blotting

EVs and whole cell pellets were lysed in 100 μL of radio immunoprecipitation assay (RIPA) buffer, containing 0.5 mM dithiothreitol (DTT), 1 mM phenylmethylsulfonyl fluoride (PMSF), and 10 μL of protease inhibitor cocktail (Sigma-Aldrich, UK). The protein concentration of lysates was measured by bicinchoninic acid assay (Pierce, Thermo Fisher). A measure 5 μg of each sample was separated by electrophoresis on 4–12% Bis-Tris gels (Nupage^™^, Thermo Fisher) at 180 V for 45 min. Proteins were semi-dry transferred to polyvinylidene fluoride membranes, blocked for 1 h at room temperature in buffer containing 5% FCS and 5% skimmed milk powder in Tris-buffered saline (TBS). Membranes were stained overnight at 4°C with primary antibody (Supplementary Table S1), which was diluted in TBS, and then washed 3 × 15 min in TBS-Tween (0.1%, Thermo Fisher) before secondary staining for 1 h with HRP-conjugated secondary antibodies. Western HRP substrate chemiluminescent solution (Millipore, MA, USA) was used to reveal bands, which were visualised using Fuji X-ray film (Thermo Fisher Scientific). The density of bands present was quantified using ImageJ software (National Institutes of Health, Bethesda, MD).^1^

### RNA isolation

RNA was isolated from Müller cell pellets using the RNeasy total RNA isolation kit (Qiagen) as per the manufacturer’s instructions. RNA was also isolated from EV for miRNA sequencing and qPCR validation using the Total Exosome RNA Isolation Kit (Invitrogen^™^) as per the manufacturer’s instructions. In order to ensure that only EV-encapsulated RNAs were analysed, intact EVs in solution were treated with RNase A enzyme (100 U/mL) (Invitrogen^™^, MA, USA) for 15 min at 37°C before the addition of RNase inhibitor (1 U/mL). To confirm that RNA was internalised, an equivalent quantity of EV was pre-treated with a membrane-disrupting detergent (Triton X-100, 0.1% v/v), vortexed briefly, and incubated for 15 min at room temperature prior to the addition of the RNase enzyme. A second control consisting of 1 μg of total cellular RNA recovered from MIO-M1 cells was diluted in an equivalent volume of PBS before receiving the RNase enzyme treatment. Samples were then re-pelleted by ultracentrifugation at 100,000 × *g* for 90 min at 4°C prior to miRNA isolation. The RNA was purified and analysed using the methods described above. RNA was quantified by a NanoDrop UV spectrophotometer (Nanodrop-1000, Thermo Scientific) before storage at −80°C.

### Size characterisation of RNA isolated from EV and whole cell samples

Prior to sequencing, whole cell and EV RNAs were assessed by electrophoresis using the 2200 TapeStation system (Agilent), using High Sensitivity RNA ScreenTape (5067–5579), High Sensitivity RNA ScreenTape Sample Buffer (5067–5580) and High Sensitivity RNA ScreenTape Ladder (5067–5581). The range of total RNA input was 500–10,000 pg/μL, and the total RNA integrity was estimated using the software RNA Integrity Number (RIN). ScreenTapes were analysed using the TapeStation Analysis Software. Peaks were identified using Agilent’s proprietary statistical calculations integrated into the software (picogram per microliter).

### Sequencing of microRNAs

The preparation of libraries and sequencing was conducted by the UCL genomics service at the UCL Institute of Child Health (UCL, London, UK). miRNA libraries were prepared using the QIAseq miRNA library kit (Qiagen, Manchester, UK) according to the Illumina small RNA sample preparation protocol. Six libraries were generated using 250 ng of total RNA from three MIO-M1 whole cell samples at passages 22, 24, and 26, and from three EV samples isolated from MIO-M1 cells at similar passage numbers (passages 23, 25, and 27). Since previous studies have shown that the MIO-M1 cell line is stable throughout several passages (Limb et al., 2002), it was expected that this approach would ensure that the samples were true biological replicates. The library pool was sequenced in a NextSeq 500 sequencing instrument (Illumina, Inc., San Diego, CA) according to the manufacturer’s instructions in a single-end read fashion with a read length of 75 bases and an approximate depth of 10–15 million reads per sample. Raw data were demultiplexed and text-based FASTQ files stored sequence, and quality scores for each sample were generated using the software bcl2fastq (Illumina Inc., San Diego, CA).

The quality of the generated reads was confirmed using the FASTQC (version 0.11.9) quality control tool (Andrews, 2010). Adapters were trimmed using Cutadapt (Martin, 2011), and raw reads were then mapped to the miRbase database (release 21) (Kozomara et al., 2019) using the Bowtie2 read alignment tool (Langmead and Salzberg, 2012), before summarisation using the featureCounts programme (Liao et al., 2014). Differential expression (DE) analysis was conducted using the R-Bioconductor statistical package DESeq2 (version 1.10.1) (Love et al., 2017).

To identify DE miRNAs with high confidence, counts were pre-filtered to remove lowly expressed transcripts (≥5 CPM in at least 50% of the samples), and filtered counts were then normalised by the DESeq2 internal procedure. The false discovery rate (FDR) was controlled at 5% using the Benjamini–Hochberg method for each pairwise comparison, and miRNAs were considered differentially abundant when the log2-fold change was ≥2.5 or ≤ −2.5.

### Bioinformatic analyses of EV microRNAs

The MiRWalk ‘microRNA-gene target’ tool was used to find predicted gene targets for each of the 20 most highly abundant miRNAs (Sticht et al., 2018). Interactions were filtered for those that intersected both the Targetscan and miRDB prediction software, were present in 3 UTR of target genes, and were associated with a confidence score of ≥95%. Gene set enrichment analysis was then conducted using the Kegg pathway geneset, and regulation of pathways was considered valid if associated with a BH-adjusted value of *p* of <0.01 (Kyoto Encyclopedia of Genes and Genomes) (Kanehisa et al., 2023). The miRTarbase (V8) database of experimentally supported miRNA/mRNA interactions was used to further investigate miRNA abundance in Muller glial cell EV. Interactions were filtered to include only those that had been validated in low-throughput experiments in human experimental models.

### Quantitative real-time PCR for the validation of microRNA sequencing

For the validation of miRNA of interest in EV and whole cell samples, a total of 3 μL of synthetic *Caenorhabditis elegans* cel-miR-39 (Norgen Biotek Corporation, Canada) was added to each sample as a spike control prior to extraction of RNA (Selth et al., 2012). RNA samples recovered from EV were reverse-transcribed using the microScript microRNA:cDNA Synthesis Kit as per the manufacturer’s instructions (Norgen Biotek Corporation). SYBR-based qRT-PCR was performed using miRNA-specific primers designed using the mIRPrimer2 (Busk, 2014) software (Supplementary Table S2). For the quantification of gene expression, real-time PCR was conducted using the SYBR Fast Universal qPCR Master Mix (Applied Biosystems^™^) on a QuantStudio^™^ 6 Flex Real-Time PCR System (Applied Biosystems^™^). For the quantification of the cel-miR-39 internal control transcript, a gene-specific forward primer was used in combination with a universal reverse primer (Norgen Biotek Corporation, Canada). At the end of the run, the threshold cycle (Ct) value was exported from the QuantStudio^™^ 6 Flex Real-Time PCR software (v1.1). Data were analysed using the 2^−ΔΔCt^ method to quantify fold change relative to the expression of cel-miR-39 control in each sample (Livak and Schmittgen, 2001). The correlation in expression abundancies between RNAseq and qRT-PCR was determined using Pearson’s correlation coefficient.

## Results

### Characterisation of Müller glia-derived EV

**Figure 1:**
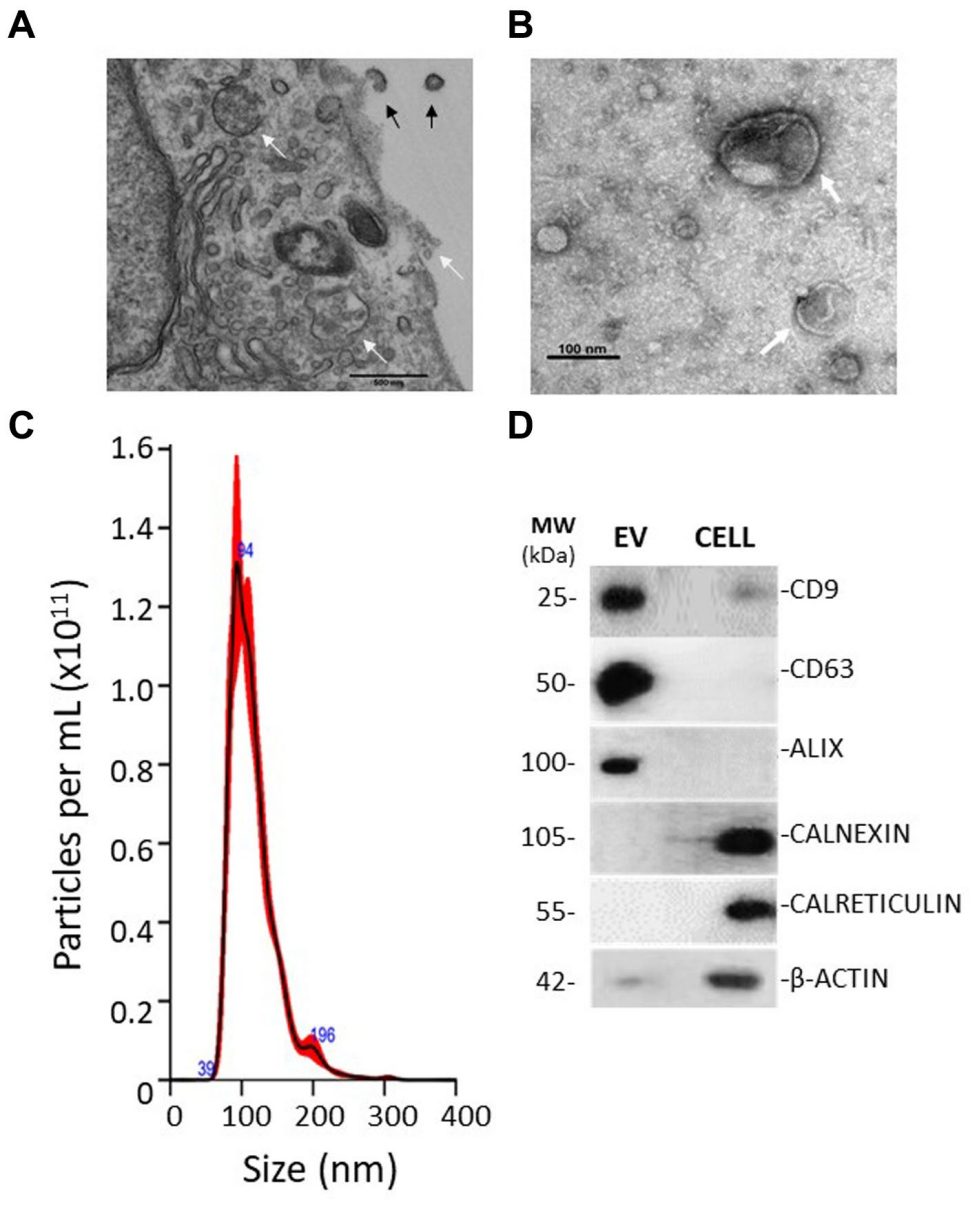
Electron micrographs show evidence of EV genesis and morphology. **(A)** TEM micrograph of a MIO-M1 cell in cross-section. Indicated by black arrows are microvesicles being shed from the cell membrane. White arrows identify the presence of multi-vesiculated late endosomes and their fusion with cell membranes. Exosomes appear to be released. **(B)** TEM micrograph of an EV preparation derived from Müller glia displaying spherical structures of variable diameters that exhibit a double-membrane morphology (white arrows). **(C)** Nanoparticle tracking analysis shows the distribution of particles present in EV preparations obtained. EV ranged from approximately 50–250 nm in diameter. **(D)** Representative Western blots demonstrating EV expression of the endocytic membrane markers CD9, CD63, and ALIX and the absence of endoplasmic reticulum and cytoskeletal protein markers when compared with whole cell lysates. Blots are representative of three different experiments.

Transmission electron microscopy examination of cells in culture showed EV particles being shed from the MIO-M1 cell surface (Figure 1A), whilst a similar analysis of purified EV showed their characteristic morphology with double membrane, consistent with previous reports of endosome-derived exosomes (Figure 1B). Nanoparticle tracking analysis (NanoSight, Malvern) indicated that the majority of the particle population was sized between 50 and 200 nm (mean 112 +/− 23 nm) (Figure 1C). Western blotting analysis of EV protein lysates demonstrated the presence of abundant proteins characteristic of endosome-derived exosomes. These included the tetraspanins CD9 and CD63, as well as programmed cell death 6-interacting protein (ALIX). These proteins were not detected in whole cell lysates when testing equivalent protein concentrations to those of the EV tested. In contrast, proteins that characterise the endoplasmic reticulum, including calnexin, calreticulin, and β-actin, were not observed in EV samples but were abundant in equivalent protein concentrations of whole cell lysates (Figure 1D).

**Figure 2:**
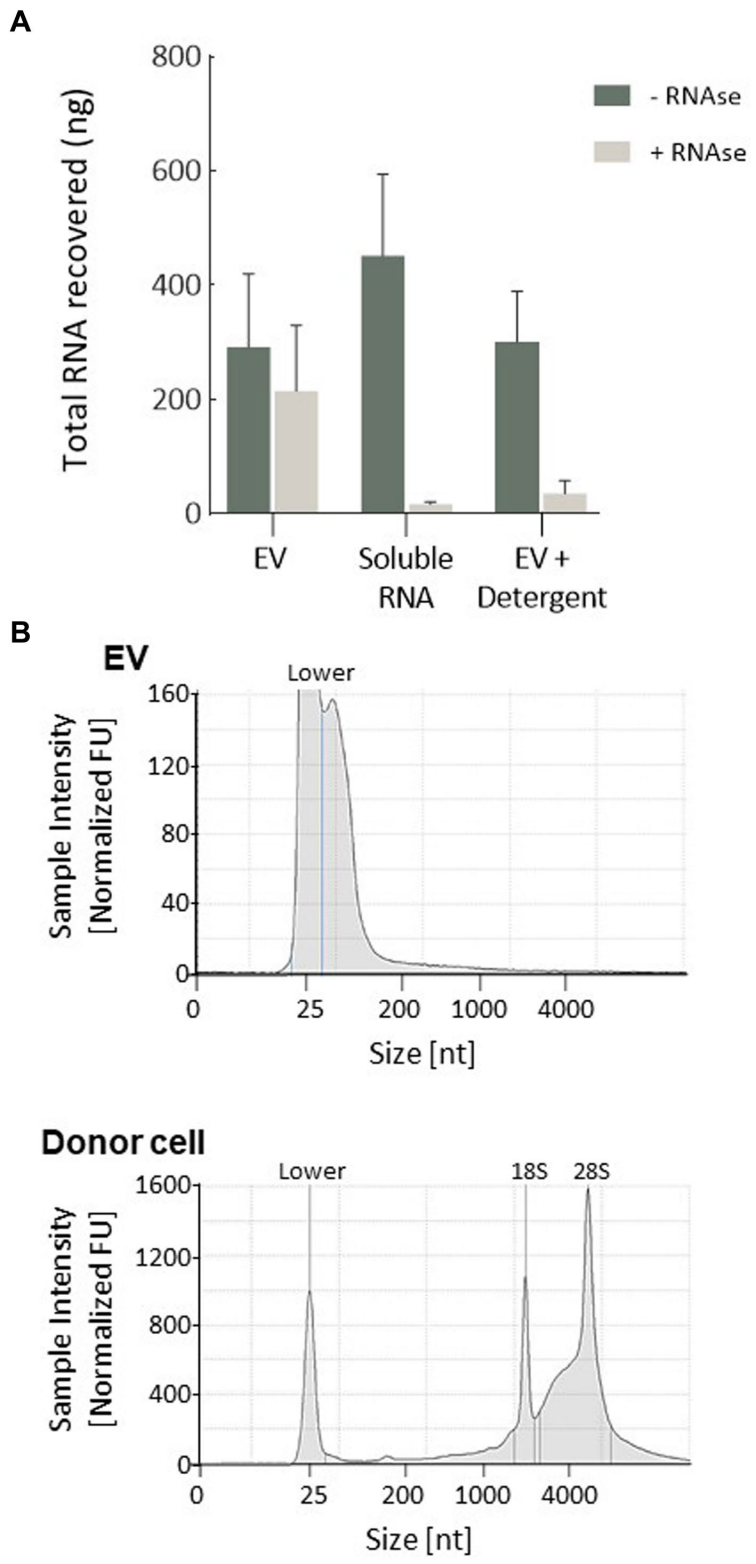
Characterisation of RNA isolated from EV. **(A)** EVs were enriched in RNA molecules that were encapsulated within the vesicle lumen, as demonstrated by protection against RNAse degradation. Plots represent the mean of three replicates refined from equivalent quantities of purified EV. Intact EV treated with RNase showed no difference in RNA degradation compared to control EV, whilst 1 μg of cellular RNA or EV receiving a membrane-disrupting detergent (Triton X-100, 0.1%) was completely degraded. **(B)** Representative electropherograms indicating small-RNA enrichment (< 200 nucleotides) in isolated EV, which lacked long-coding RNA. Whole MIO-M1 cells had a characteristic RNA profile comprised of both small non-coding and large-coding species, with peaks representing ribosomal RNA subunits (18S and 28S).

As shown in Figure 2A, levels of RNA isolated from EV were not significantly reduced by treatment with RNAse A, whilst treatment of soluble RNA obtained from whole cell lysates significantly degraded unprotected RNA. In addition, EV membrane disruption with TRIzol to release their RNA caused degradation of the released RNA by RNAse treatment. These observations indicate that nucleic acids were internalised within vesicle lumens rather than merely co-precipitating during the collection process. Assessment of total RNA samples recovered from EV by the Agilent Bioanalyzer indicated that these particles lacked 18 and 28 ribosomal RNA subunits and were instead enriched in small non-coding RNAs <100 nucleotides in length (Figure 2B).

### Sequencing of RNA contained in MIO-M1 cells and their secreted EV

**Figure 3:**
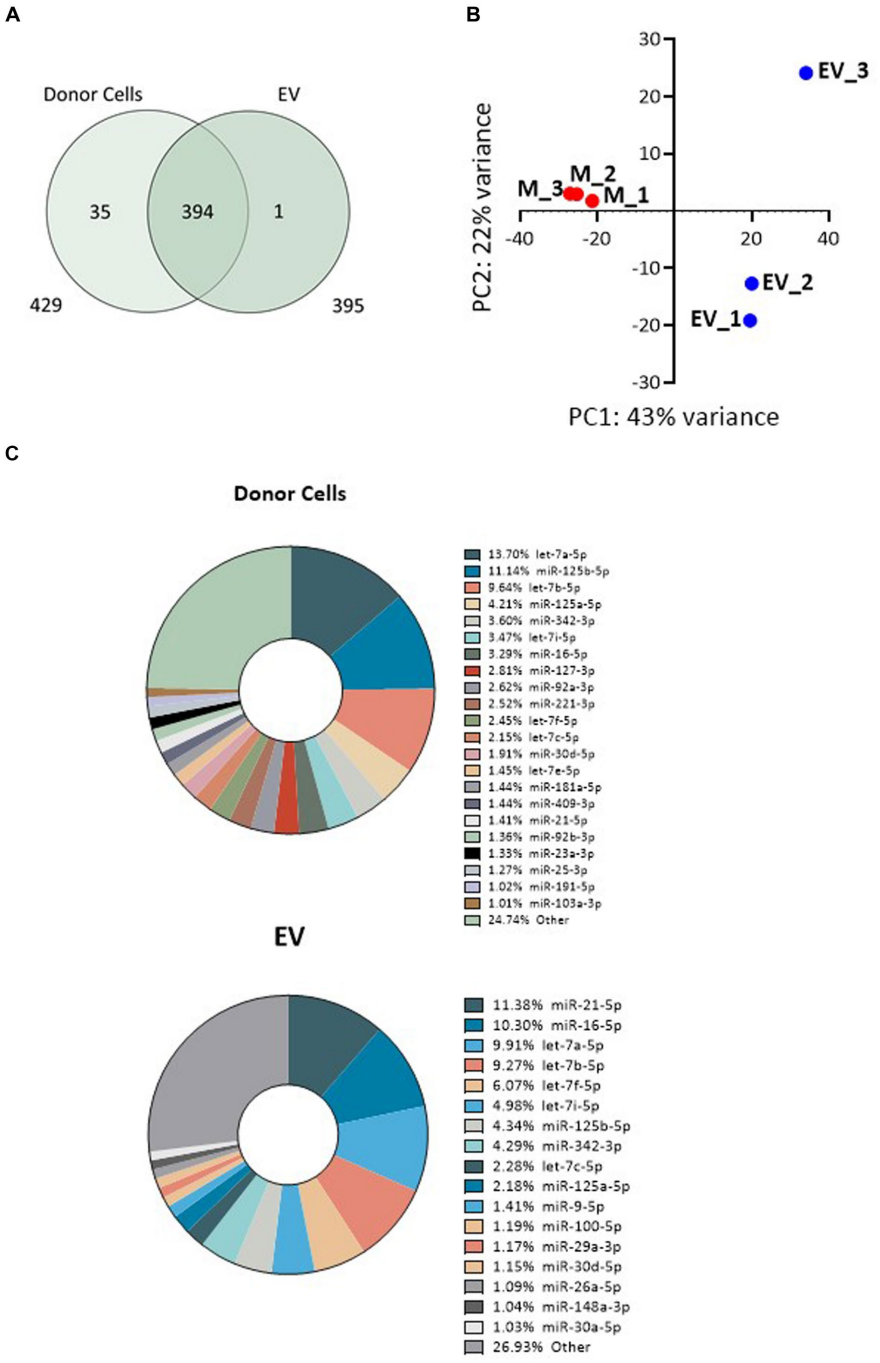
Variability of miRNA expression between whole MIO-M1 cells and secreted EV miRNA libraries. **(A)** Venn diagram displaying the unique and overlapping miRNAs identified in whole cells and in isolated EV. **(B)** Principal component analysis (PCA) displays variance of mapped miRNA reads between the six individual libraries. “M” refers to whole MIO-M1 cells and “EV” to their secreted EV. **(C)** Pie charts showing relative miRNA abundance as determined by high-throughput miRNA RNA sequencing. miRNAs representing greater than 1% of the total read count are indicated for EV and whole MIO-M1 cell samples.

Illumina RNA sequencing was used to profile the miRNA signatures of Müller cells from the MIO-M1 cell line, as well as those from EV secreted by these cells in culture. Raw reads for all six libraries analysed passed FastQC quality control and were aligned to the miRbase reference library (V22). For the three samples recovered from donor cells, a mean of 1.88e6 reads were assigned to miRNA, an alignment rate greater than 95% (see Supplementary Table S3). Samples sequenced from the three EV sources had a slightly lower rate of alignment (mean 89.1%), but a comparable number of miRNA-assigned reads (2.13e6). After normalisation (EdgeR: log2 (CPM + 4)), a total of 430 miRNA passed filtering to remove lowly expressed annotations (≤5 counts per million across three replicate libraries, as presented in Supplementary Table S4). Of these, the vast majority (429) were present in whole cells, whilst fewer (395) were detected within a sample of secreted EV. A total of 394 were therefore common between both sample sources (Figure 3A). Principle component analysis (PCA) and clustering were used to assess intra-sample and inter-sample variability. Tight clustering of whole cell samples 1, 2, and 3 indicated a high level of in-sample consistency between the replicates (Figure 3B), and considerable distance was observed between these and the three EV-derived samples, suggesting that the miRNA signatures of the two sources varied considerably. Greater variability was also observed within the three EV-derived samples, where sample 3 was noticeably distant from replicates 1 and 2. Whilst whole cell samples were directly lysed for miRNA isolation, the protocol for EV isolation included membrane filtration of culture supernatants followed by sequential recovery of supernatants from successive centrifugation steps. This process undoubtedly may give more opportunities for variations in miRNA isolation. These variations may also reflect the relative abundance of miRNA recovered from whole cells when compared to the smaller quantities recovered from EV. However, our data suggest that whilst there is some variation between the EV libraries, this is principally found amongst the lowly expressed miRNAs. When the 75% most abundant miRNAs are considered, the coefficient of correlation in expression between EV3 and EVs 1 and 2 is 0.84 and 0.85, respectively. This compares to correlations of <0.75 when EV samples are compared with whole cells.

**Figure 4:**
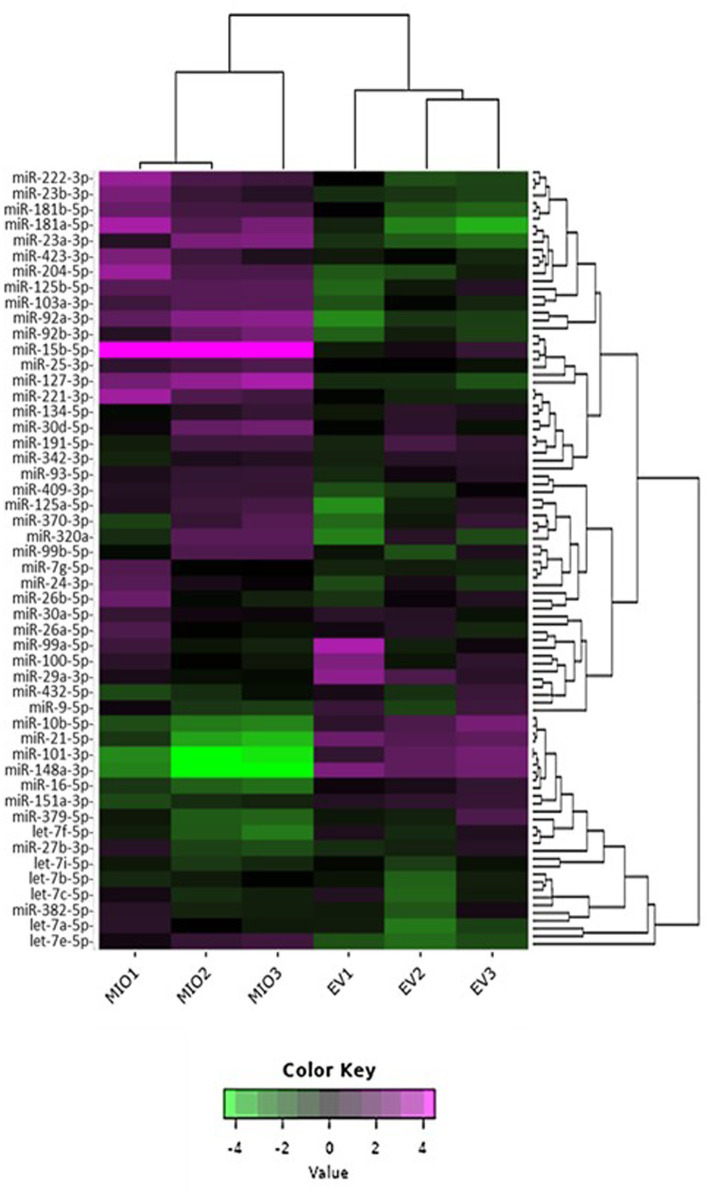
2D Heatmap presenting the variability of miRNA expression between sample libraries. The 50 most variable miRNA species are shown for comparison. EV samples are labelled “EV,” whilst those from whole cells are labelled “MIO.” Hierarchical clustering was performed across samples and miRNA; purple indicates high comparative abundance, whereas green denotes low comparative abundance.

To evaluate the relative distribution of miRNAs in whole cells and their exosomes, we ranked cellular and EV miRNA based on log2 counts per million (CPM), and compared the 20 most abundant species detected in each of the two sample sources (Table 1). miRs 21-5p, 125b-5p, 16-5p, 342-3p, and members of the let-7 miRNA family were amongst the most represented transcripts common to both sample types. Although there was substantial variability in their respective relative abundance, several miRNAs (highlighted in Table 1) were present only in either the list of cellular species or in the list of EV-abundant species. Figure 3C displays the most abundant miRNA in both EV and whole cells as a percentage of the total miRNA load. In EV, the most abundant miRNAs were miRs 21-5p and 16-5p which comprised 11 and 10%, respectively, of the total miRNA load present within these organelles. This signature differed substantially from that of the donor cells, where let-7a-5p was the most abundant species (13.7%), and miRs 21-5P and 16-5p represented a far smaller proportion of the total reads, 1 and 3%, respectively. The heatmap in Figure 4 shows hierarchical clustering of the 50 most variably expressed miRNA species between sample sources.

**Table 1:** Mean normalised read counts for the 20 most abundant miRNA detected in intracellular Müller glia samples, compared to those recovered from EV (data derived using the DESeq2 package).

Rank	Donor MIO-M1 cells		Secreted EV	
	miRNA	Normalised read count	miRNA	Normalised read count
1	let-7a-5p	17.24	miR-21-5p*	16.87
2	miR-125b-5p	16.98	miR-16-5p	16.66
3	let-7b-5p	16.77	let-7a-5p	16.58
4	miR-125a-5p	15.54	let-7b-5p	16.54
5	let-7i-5p	15.35	let-7f-5p	15.93
6	miR-342-3p	15.27	let-7i-5p	15.64
7	miR-16-5p	15.2	miR-342-3p	15.29
8	**miR-127-3p**	**14.98**	miR-125b-5p	15.26
9	**miR-92a-3p**	**14.83**	let-7c-5p	14.54
10	miR-221-3p	14.8	miR-125a-5p	14.14
11	let-7f-5p	14.72	**miR-9-5p**	**13.85**
12	let-7c-5p	14.66	**miR-29a-3p**	**13.6**
13	miR-30d-5p	14.26	**miR-100-5p**	**13.59**
14	**miR-181a-5p**	**14.11**	miR-30d-5p	13.42
15	**let-7e-5p**	**13.99**	**miR-26a-5p**	**13.42**
16	**miR-409-3p**	**13.99**	miR-148a-3p*	**13.41**
17	miR-21-5p	13.9	**miR-30a-5p**	**13.36**
18	**miR-92b-3p**	**13.88**	miR-221-3p	13.3
19	miR-25-3p	13.84	**miR-191-5p**	**13.03**
20	**miR-23a-3p**	**13.83**	miR-25-3p	12.98

Species unique to each sample source are highlighted in bold; species significantly differentially enriched are indicated with * (>2.5 Log2 FC, Adj-*p* = < 0.01).

### Differential expression (DE) analysis

**Figure 5:**
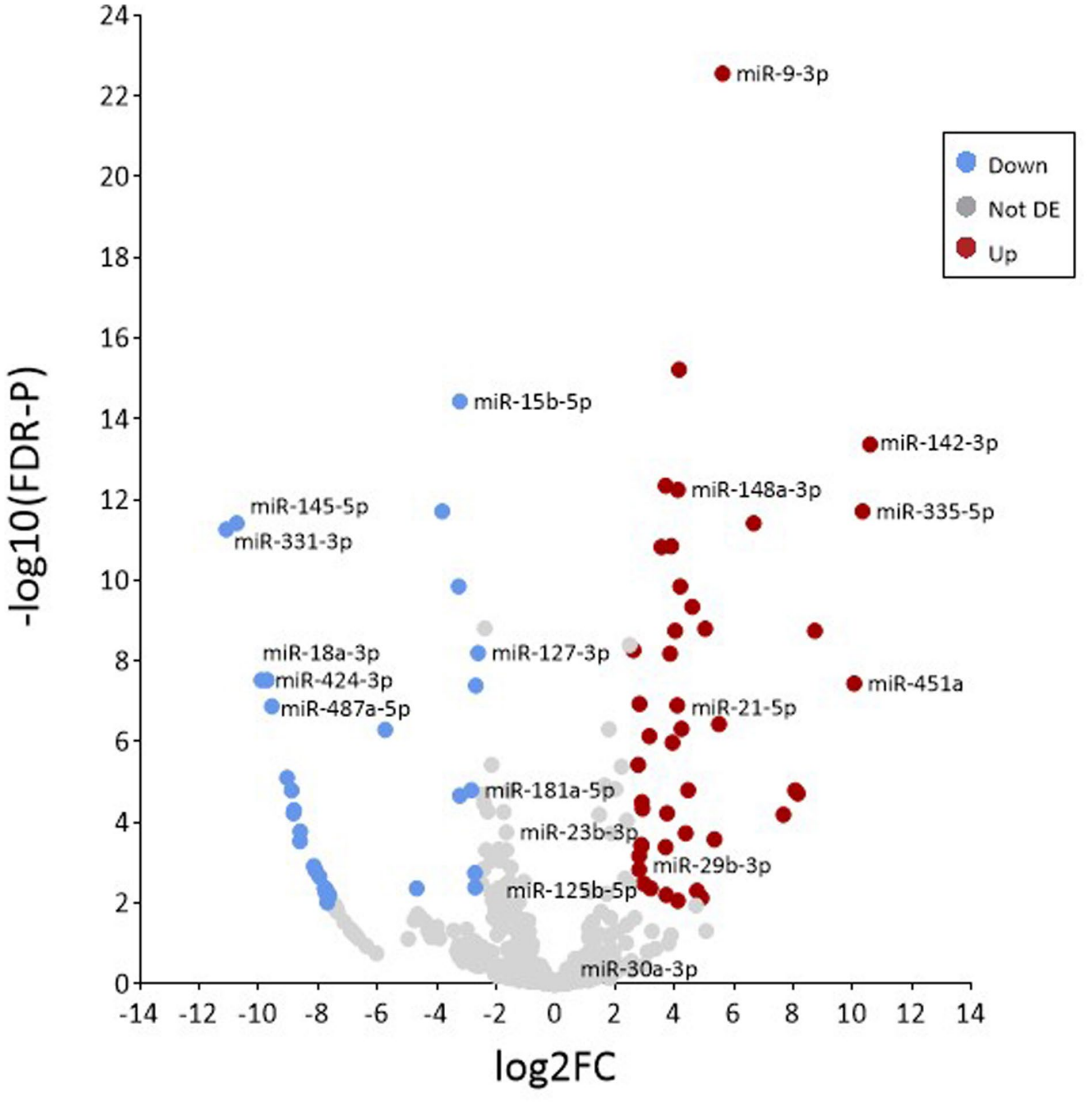
Analysis of differential enrichment. Volcano plot representing the distribution of differentially expressed miRNAs between samples based on the –log base 10 significant *p* value (<0.01) and a relative fold change >2.5 (in log base 2). Red and blue circles represent increasingly and decreasingly enriched microRNAs, respectively, when compared to isolated EV versus whole cells. Grey circles represent miRNAs that were not significantly enriched.

Differential expression analysis using the R software package DESeq2 was used to identify specific miRNAs that were expressed in EV at significantly higher abundance than would be expected under random assortment. DESeq2 uses the average expression strength of each gene across all samples as its filter criterion, and it omits all genes with mean normalised counts below a filtering threshold from multiple testing adjustments (Supplementary Table S5). A total of 77 miRNAs were found, of which 45 were upregulated and 32 downregulated in EV when compared with donor MIO-M1 cells, using a 2.5 log2-fold change and FDR-*p* < 0.01 as the cutoff threshold (Table 2). As presented in Figure 5, 45 of these DE species were found to be significantly more enriched in EV compared to donor cells. Of note were miR-142-3p, mir-335-5p, and miR-451a, which were each found to be >10 log2-fold change enriched in EV compared to donor cell sources (*p* = <0.001). In addition, two of the most highly abundant miRNAs (>1% total yield), miR-21-5p and miR-148a-3p, were also found to be DE enriched in EV (Figure 5). In contrast, 31 transcripts were found to be comparatively depleted in EV, including miR-487a-5p, miR-18a-3p, miR-424-3p, miR-145-5p, and miR-331-3p which were each detected at a factor < −9 log2-fold in EV compared to whole Muller glial cell samples.

**Table 2:** Full list of 77 DE miRNA identified by DESeq2 analysis of EV and donor MIO-M1 cells.

Number	Up		Down	
	miRNA	Log2 FC	miRNA	Log2 FC
1	miR-142-3p	10.58	let-7d-5p	−1.58
2	miR-335-5p	10.33	miR-23b-3p	−1.66
3	miR-451a	10.05	miR-221-3p	−1.66
4	miR-126-5p	8.72	let-7e-5p	−1.75
5	miR-1246	8.15	miR-222-3p	−1.86
6	miR-374a-3p	8.06	miR-92b-3p	−1.92
7	miR-19a-3p	7.67	miR-409-5p	−2.16
8	miR-590-3p	6.67	miR-204-5p	−2.16
9	miR-9-3p	5.62	miR-149-5p	−2.29
10	miR-137	5.5	miR-210-3p	−2.35
11	miR-301a-3p	5.34	miR-323b-3p	−2.39
12	miR-1	5.03	miR-423-3p	−2.4
13	miR-136-3p	4.6	miR-107	−2.46
14	miR-3613-5p	4.46	miR-127-3p	−2.61
15	miR-4516	4.37	miR-92a-3p	−2.69
16	miR-203a	4.23	miR-181a-5p	−2.84
17	miR-450b-5p	4.19	miR-15b-5p	−3.22
18	miR-411-5p	4.15	miR-484	−3.23
19	miR-148a-3p	4.11	miR-493-5p	−3.26
20	miR-582-3p	4.09	miR-299-5p	−3.82
21	miR-889-3p	4.03	miR-130b-5p	−5.74
22	miR-122-5p	3.93	miR-1226-3p	−8.6
23	miR-376a-3p	3.89	miR-379-3p	−8.6
24	miR-29c-3p	3.85	miR-4521	−8.81
25	miR-6087	3.75	miR-185-3p	−8.82
26	miR-7641	3.7	miR-129-5p	−8.89
27	miR-101-3p	3.7	miR-29c-5p	−9.04
28	miR-382-3p	3.56	miR-487a-5p	−9.55
29	miR-126-3p	3.15	miR-18a-3p	−9.71
30	miR-192-5p	2.92	miR-424-3p	−9.91
31	miR-199a-3p	2.9	miR-145-5p	−10.74
32	miR-376c-3p	2.88	miR-331-3p	−11.08
33	miR-340-5p	2.88		
34	miR-21-5p	2.82		
35	miR-29b-3p	2.81		
36	miR-628-5p	2.78		
37	miR-143-3p	2.62		
38	miR-10b-5p	2.49		
39	miR-660-5p	2.4		
40	miR-320c	2.22		
41	miR-182-5p	2.01		
42	miR-183-5p	1.87		
43	miR-10a-5p	1.79		
44	miR-381-3p	1.65		
45	miR-16-5p	1.47		

### Pathway analyses of EV-abundant microRNA

In order to gain insight into the potential functions of Müller glia EV, gene set enrichment analysis for the 20 most abundant miRNAs detected in Müller-secreted vesicles was conducted using the miRWalk microRNA-gene target tool, which predicted 3UTR binding interactions with 1352 target genes. The 20 Kegg pathways predicted to be most significantly interacted by EV-miRNA are listed in Table 3 and were found to comprise principally signalling cascades involved in cell cycle control, longevity, and survival, as well as cell-matrix adhesion regulation. These included numerous interactions with genes involved in growth factor signalling, neuron elongation, and inhibition of apoptosis: MapK, PI3K-Akt, and axon guidance, which may represent plausible targets for miRNA interference of gene expression to influence the survival and regeneration of retinal neurons. In addition to these pathways, gene networks principally associated with cancer formation and progression were observed, which is not surprising in immortalised cells such as the MIO-M1 cell line (Table 3).

**Table 3:** The output of miRwalk gene set enrichment analysis for the twenty most abundant miRNA present in Müller-EV.

Number	Kegg orthology group identifier	Gene count	miRNA count	BH adjusted *p*-value
1	Pathways in cancer (hsa05200)	61	16	0.01
2	MAPK signalling pathway (hsa04010)	55	17	0.00
3	PI3K-Akt signalling pathway (hsa04151)	52	15	0.00
4	Human papillomavirus infection (hsa05165)	42	15	0.01
5	Focal adhesion (hsa04510)	38	15	0.00
6	Ras signalling pathway (hsa04014)	32	17	0.02
7	Proteoglycans in cancer (hsa05205)	31	13	0.01
8	Axon guidance (hsa04360)	29	15	0.01
9	Transcriptional misregulation in cancer (hsa05202)	28	14	0.01
10	Neurotrophin signalling pathway (hsa04722)	20	13	0.01
11	EGFR tyrosine kinase inhibitor resistance (hsa01521)	18	14	0.01
12	Longevity regulating pathway (hsa04211)	18	12	0.01
13	ECM-receptor interaction (hsa04512)	18	9	0.01
14	Small cell lung cancer (hsa05222)	18	11	0.01
15	Hypertrophic cardiomyopathy (hsa05410)	17	15	0.01
16	Dilated cardiomyopathy (hsa05414)	17	16	0.02
17	p53 signalling pathway (hsa04115)	16	11	0.01
18	Polycomb repressive complex (hsa03083)	16	14	0.01
19	Glioma (hsa05214)	15	11	0.01
20	Other types of O-glycan biosynthesis (hsa00514)	11	11	0.02

The 20 most significantly regulated Kegg pathways are shown, ranked according to the number of predicted gene interactions. Interactions were filtered for those that intersected both the Targetscan and miRDB prediction software, with a confidence score of ≥95%.

**Figure 6:**
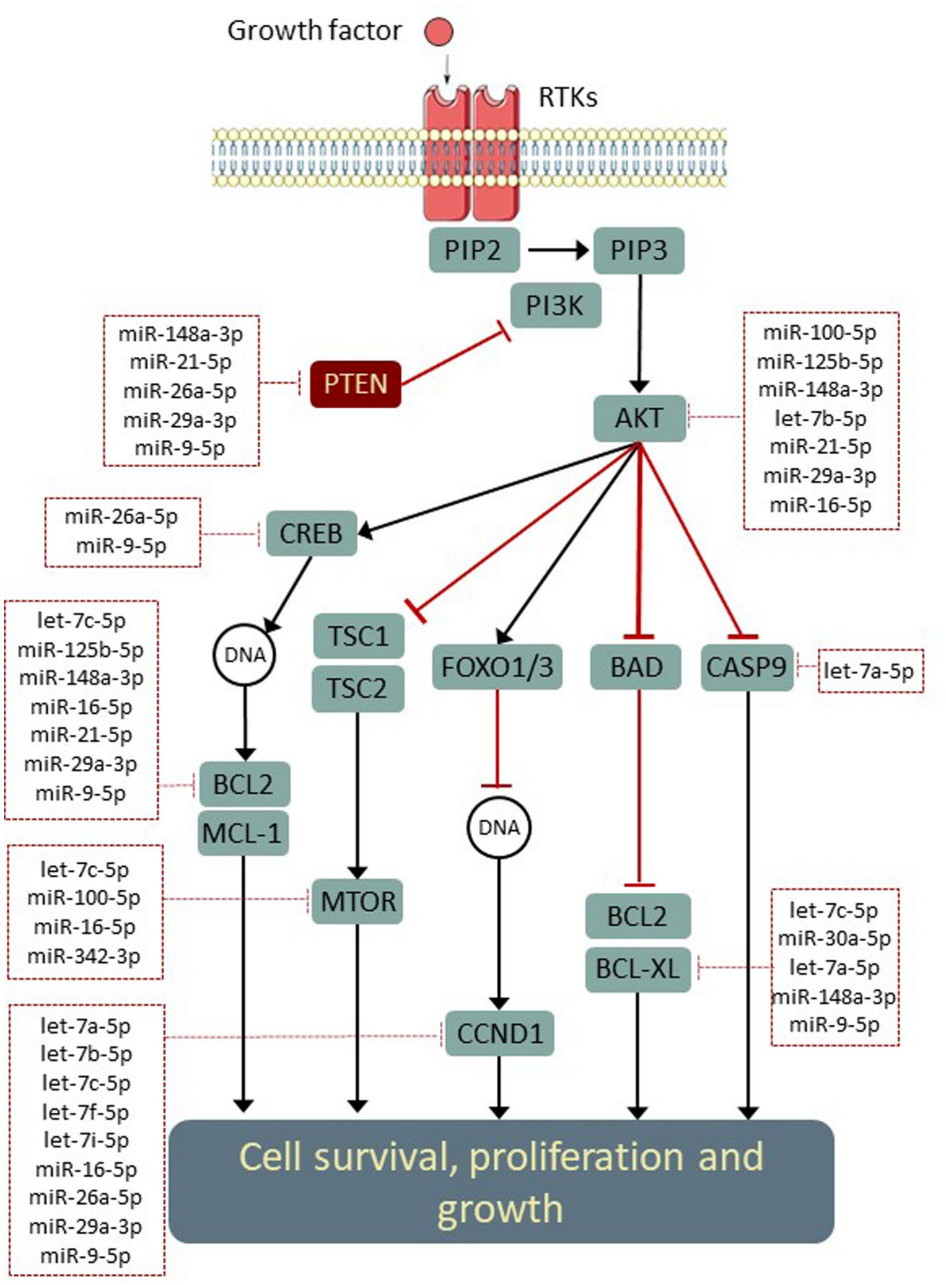
Opportunities for the post-transcriptional modulation of genes in neuroprotective signalling pathways. Simplified schematic (adapted from KEGG: hsa04151) indicating experimentally validated regulatory interactions between Müller miRNA (within dotted boxes) and key genes within neuroprotective signalling networks. Interactions were identified using (Tarbase v8) and filtered to include only validations in human models.

Since several of these networks have been previously associated with neuroprotective activity, we conducted further investigations in order to evaluate the influence of abundant Müller EV-miRNA in these pathways. The miRTarbase (V8) database of experimentally validated microRNA target interactions was scanned for low-through relationships between Müller-derived EV miRNA and specific genes involved in pathways of interest (Figure 6). These observations highlight several important mediators of cell functions, including the MAPK and PI3K-AKT signalling pathways, which are known to interact with molecules that regulate apoptosis, cell growth, and neuroprotection, such as PTEN, SOCS3, and mTOR (Figure 6).

### Validation of expression profiles

**Figure 7:**
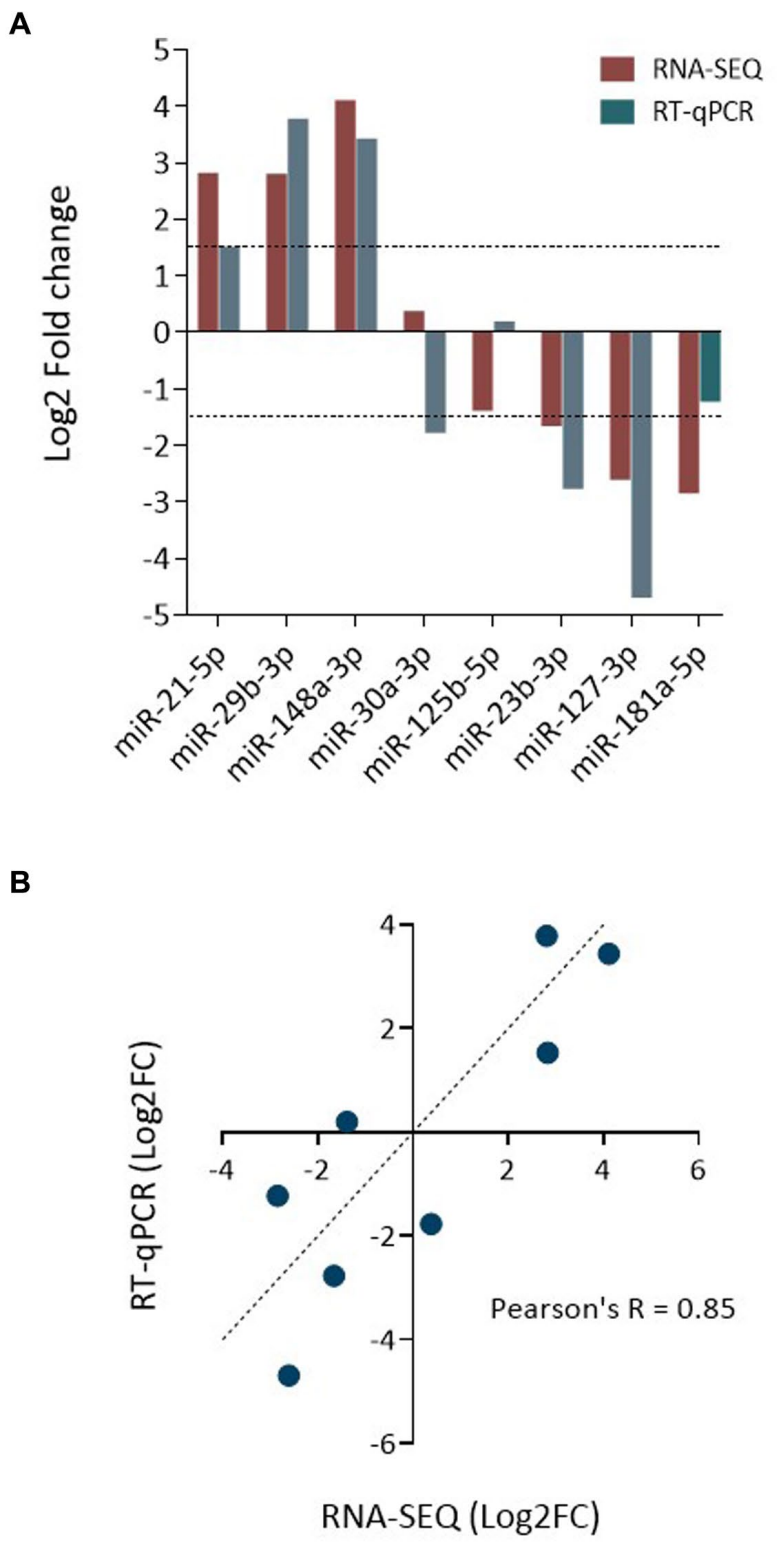
Validation of data obtained by miRNA sequencing. **(A)** Bar graph compares the relative enrichment (log2-fold change) of select miRNA between EV and donor cell samples, as determined by RNA-seq and qRT-PCR. The expression of three EV-enriched and three EV-depleted miRNAs was selected for validation, as were two control miRNAs. **(B)** Correlation analysis plot of miRNA expression as log2-fold change as determined by qPCR and RNA-seq. Shown is the linear regression analysis and Pearson’s correlation coefficient (Pearson’s *R* = 0.85; *R*^2^ = 0.73; **p = 0.007).

In total RNA recovered from EV and whole MIO-M1 cells, the relative expression of three over-represented miRNA (miR-21-5p, miR-29b-5p, and miR-148-3p) and three under-represented miRNAs (miR-125b-5p, miR-127-3p, and miR-181a-5p) was validated by real-time quantitative PCR (RT-qPCR) relative to the detection of an exogenous spike-in control (*cel-miR-39*) (Figure 7A). In addition, miR-30a-3p and miR-125B-5p, which were not determined to be DE between the two samples, were also validated. Each of the transcripts investigated was found to be expressed in both sample sources, and their relative enrichment profiles were comparable with the results of the DE analysis, with the exception of miR-181a-5p, which was not found to be downregulated in EV as compared to whole cell samples (−1.38 Log2FC). Comparison of fold changes between RNA seq and qPCR data revealed a significant correlation (Pearson’s *R* = 0.85; **p = 0.007, Figure 7B). This data confirms the validity of the sequencing analysis undertaken for miRNA obtained from both EV and whole cells.

## Discussion

The present study provides the first analysis of the miRNA profile expressed in EV secreted by human Müller glial cells. Sequencing revealed a significant differential enrichment of a subset of transcripts in EV compared to the whole Müller cells from which they originated and showed substantial variability between the most abundant species in both preparations. Pathway analysis indicated that the most highly expressed miRNA transcripts observed in Müller-derived EV have experimentally validated gene targets within signalling pathways associated with cell growth, neuroprotection, and protection from apoptosis (Saiijilafu et al., 2013; Zhang et al., 2014; Levin et al., 2022).

Key to the potential neuroprotective function of Müller-derived EV are numerous observations in the literature that the molecular cargos of secreted vesicles are specific to their cellular origin (Mead and Tomarev, 2017; Ching et al., 2018). In addition to the established neuroprotective and metabolic functions of Müller glia, these cells also represent a multipotent adult stem cell population in the retina, and it is logical that their secreted EVs contain cargoes that reflect both of these characteristics. It was therefore of interest that many of the most highly expressed miRNAs detected in EV isolated from Müller glia have been previously detected in vesicles released by various adult stem cells. For example, our data indicated that miR-21-5p comprised 11.38% of the total read count in EV secreted by Müller cells, and it has also been detected in bone marrow-derived mesenchymal stem cell (BMSC)-secreted EVs where its transfer to target cells is thought to be responsible for the prevention of nucleus pulposus cell apoptosis and reductions in intervertebral disc degeneration *in vivo* (Cheng et al., 2018). Similarly, miR-16-5p, another Müller EV-enriched miRNA (10.3%), is also abundant in BMSC-derived vesicles and has been associated with diverse therapeutic outcomes in rodent, including spinal cord injury repair (Tian et al., 2022), enhanced wound healing (Yan et al., 2020), and amelioration of diabetic nephropathy through podocyte protection (Duan et al., 2021).

We also found similarity between the most abundant miRNA species contained in EV secreted by human Muller cells in culture and those previously reported to be specific to primary Müller glia in the rodent retina: miR-16-5p, let-7a, let-7c, miR-125b-5p, miR-125a-5p, miR-9-5p, miR-100-5p, and miR-30d-5p (Wohl and Reh, 2016). It was observed that 34% of the total number of miRNA reads sequenced from our whole cell samples were assigned to members of the let-7 family, which were equivalently enriched in their secreted EV (32%). Our data are, therefore, in agreement with previous findings suggesting that let-7 miRNAs are constitutively expressed by primary Muller glial cells in the mouse and the fish (Ramachandran et al., 2010; Wohl and Reh, 2016). Indeed, let-7 is critical to Müller cell function in the retina, playing a role in their proliferation in the mouse (Yao et al., 2016), and being essential to the process by which these cells are able to regenerate damaged neural tissue in the zebrafish via regulation of Shh signalling (Kaur et al., 2018). The fact that Müller glia appear to be exporting these let-7 family members as well as other Müller-essential miRNA species in such significant quantities may give new insight into their role in homeostatic extracellular signalling within the retina. Our previous study has shown very strong similarities between retinal organoid-derived Müller cells and the MIO-M1 cell line (Eastlake et al., 2021). Since only the MIO-M1 cell line was used in the present study, it will be important to investigate whether EV from Müller cells derived from other sources expresses a similar profile of miRNAs.

The present study also provides further evidence for the preferential enrichment of certain miRNA transcripts in EV compared to their cells of origin. Previous publications have demonstrated that in certain instances, short sequence motifs over-represented in certain miRNAs appear to control sorting into exosomes via interactions with RNA-binding proteins like hnRNPA2B1 (Villarroya-Beltri et al., 2013) and SYNCRIP (Santangelo et al., 2016). It was observed that more than 22% of the miRNA species present in Müller EV were significantly differentially enriched when compared with the whole cells. Whether these species are selected and packaged for export to play a specific role in extracellular signalling processes or whether certain species are simply purged via EV as part of internal post-transcriptional regulation is still unclear. Whilst validation of miRNA by quantitative RT-PCR was undertaken with only six of the DE miRNAs, we recognise that increasing the number of DE miRNAs chosen for validation would increase the power of our validation experiments. However, we believe that the six DE miRNAs selected, in addition to the two controls, are sufficient to verify the results. The number of genes validated is in line with the numbers of miRNAs used in comparable studies involving miRNA sequencing of exosome-derived samples (Huang et al., 2018; Nozaki et al., 2018; Wu et al., 2020).

Although our data indicate that Müller EVs are associated with more than 395 different miRNA species, just 6 of these accounted for more than 50% of the total number of assigned reads: miR-21-5p, miR-16-5p, let-7a-5p, let-7b-5p, let-7f-5p, and let-7i-5p. Whilst further studies are required to identify the exact contribution of individual miRNA or combinations of miRNA needed for retinal neuroprotection, it may be possible to speculate that specific miRNAs present in very high quantities are likely responsible for any physiological effects observed on recipient cells. As one of the most abundant (11%) species detected in EV released by Müller glia, miR-21-5p makes an intriguing candidate that may exert neuroprotective functions. Despite associations with this miRNA as a biomarker in various cancers (Huang et al., 2013), several studies have ascribed a potential therapeutic role to this miRNA in neurodegenerative conditions of the CNS as well as the retina. Interestingly, *in vitro* overexpression of miR-21 in cortical neurons was found to suppress oxygen and glucose deprivation-induced apoptosis, whereas inhibition of endogenous miR-21 expression enhanced cell death through post-transcriptional regulation of cell death-inducing ligand FASLG (Buller et al., 2010). In a rodent model of spinal cord injury, upregulation of miR-21 was found to improve neuronal survival and promote functional recovery via the regulation of tumour-suppressor protein pdcd4 (Zhang et al., 2019), the same axis was also found to be responsible for the regulation of MSC-induced neuroprotection in an acute mouse glaucoma model (Su et al., 2017).

Analysis of the 20 most abundant miRNAs present in EV using the miRWalk target mining database indicated that the most significant interactions were predicted within biological networks and processes associated with cell growth and survival (Table 3). Of importance was a predicted modulation of the PI3K/AKT network, which has many experimentally validated interactions predicted for the most abundant EV miRNAs observed in this study, including miR-21-5p and miR-16-5p (Table 2). Whilst the PI3K/AKT pathway is active in developing tissue and is a source for complete axon regeneration in the peripheral nervous system (Saiijilafu et al., 2013; Zhang et al., 2014), its activity is suppressed in quiescent cells by the phosphoenzyme PTEN. Previous studies suggest that the direct suppression of PTEN, CASP2, and related pro-apoptotic genes by miRNA overexpression or synthetic siRNA delivery is capable of preventing the autophagy of neurons in rodent models of RGC depletion (Li et al., 2018; Thomas et al., 2021). It is therefore possible that a miRNA-induced downregulation of PTEN and subsequent activation of PI3K/AKT signalling may contribute to the neuroprotective effects attributed to Müller following whole cell transplantation studies (Singhal et al., 2012; Eastlake et al., 2019). However, further studies are needed to confirm this hypothesis. It should also be noted that the direction of modulation within these pathways is still unclear, and whilst some of the miRNAs present in Müller EV have been shown to inhibit PI3K-AKT signalling, others could promote its activity through the negative regulation of pro-apoptotic proteins such as BCL2.

Cellular survival has been demonstrated to be a fine balance between pro-apoptotic and pro-survival signals (Singh et al., 2019), and the ultimate fate of a given cell to the internalisation of Muller-EV miRNAs would likely depend upon the specific context and transcriptome of that cell as well as the quantity, stability, and availability of miRNAs received. Future studies comparing full transcriptome changes in retinal cells following exposure to Müller-EV will allow greater insight into the influence that these miRNAs may have *in situ* as well as their potential as a cell-based neuroprotective therapy.

## Conclusion

The present study constitutes the first report of the miRNA signature of EV derived from Muller glial cells. It provides an insight into this under-appreciated mechanism of intracellular communication and indicates the importance of further investigations to understand the neuroprotective secretome ascribed to these cells. The data presented here suggest that the release of vesicles abundant in miRNA, known to regulate anti-apoptotic signalling networks, may be a plausible component of the therapeutic effect observed following whole cell transplantation of Müller cells into the retina. Since previous studies also indicate that EVs released by some cells demonstrate preferential uptake by constitutive cells of the same tissue, as well as the ability to promote functions that are specific of that tissue (Zwi-Dantsis et al., 2020), it is possible that Muller glial cells may constitute a potential source for retinal-specific neuroprotective EV therapy. Furthermore, since Müller glia share the properties of radial glia in the central nervous system and are derived from the same precursors, it would be important to identify if there are any similarities between EV derived from Müller glia and EV derived from radial glia in the brain, and this merits further investigations. This would potentially open new avenues to explore new treatments for neurological disease.

## Data availability statement

The datasets presented in this study was deposited in the GEO repository, accession number GSE245286. Data can be accessed at: https://www.ncbi.nlm.nih.gov/geo.

## Author contributions

WL: Methodology, Writing – review & editing, Data curation, Formal analysis, Investigation, Validation, Writing – original draft. KE: Investigation, Methodology, Writing – review & editing, Resources. JL: Writing – review & editing, Data curation, Formal analysis, Software. NS: Writing – review & editing, Funding acquisition, Project administration, Resources. PK: Funding acquisition, Resources, Writing – review & editing, Conceptualization, Supervision. GL: Conceptualization, Resources, Supervision, Writing – review & editing, Funding acquisition, Methodology, Project administration, Visualization.

## Footnotes

^1^https://imagej.nih.gov
