# Supplementary figures and images for: MicroRNA profile of extracellular vesicles released by Müller glial cells
Source: Front Cell Neurosci. 2024 Jan 18;17:1325114. doi: 10.3389/fncel.2023.1325114 (PMC10832456; doi:10.3389/fncel.2023.1325114)

## Supplementary data 1: Original x-ray films for western blots

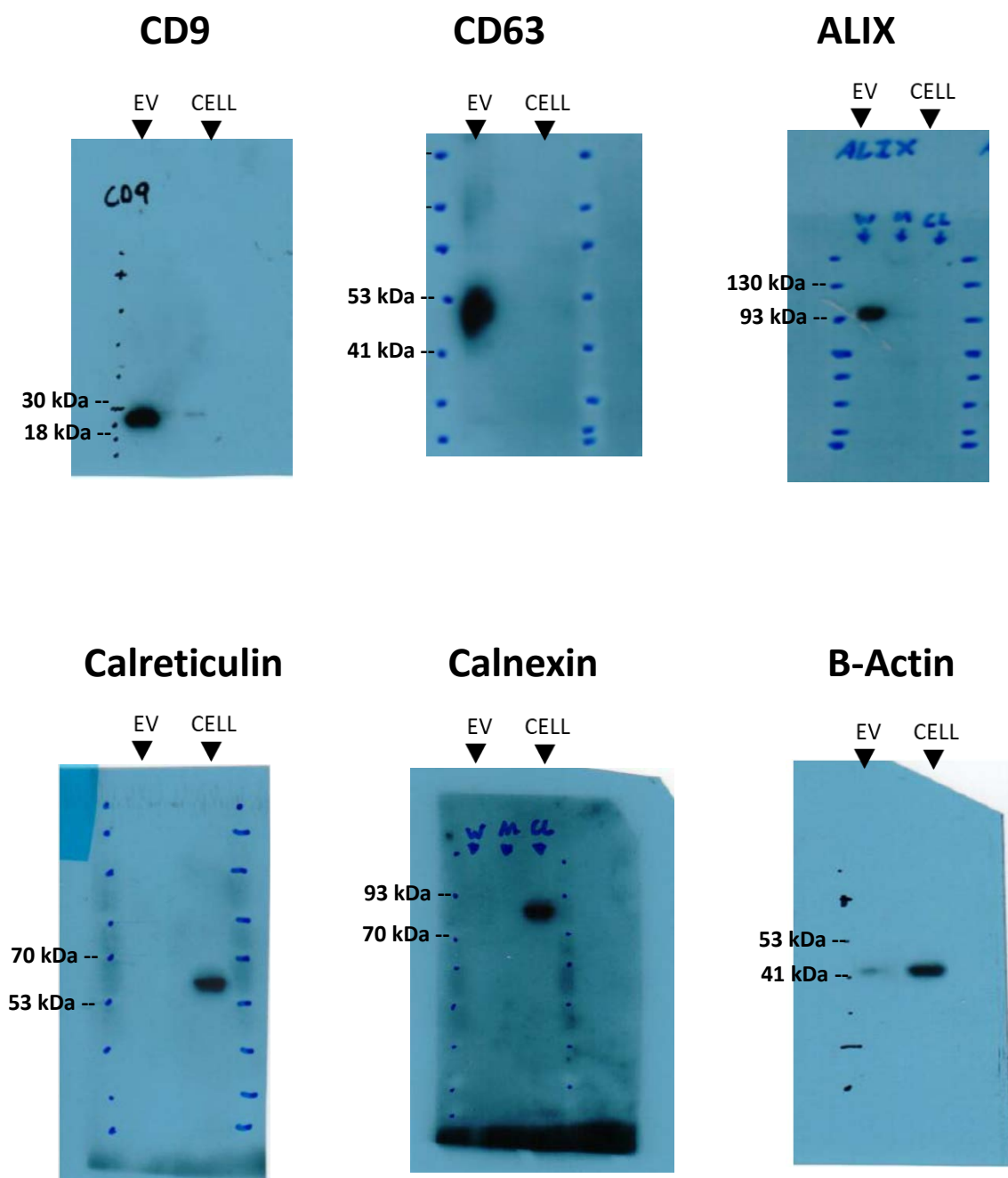

Supplement: Supplementary file 1 [file Data_Sheet_1.PDF]
